# Supplementary material for: Preliminary study on the association between lignan metabolites and CT non-destructive testing of coconut fruit at different developmental stages
Source: PeerJ. 2024 Sep 26;12:e18049. doi: 10.7717/peerj.18049 (PMC11439403; doi:10.7717/peerj.18049)
Supplement: Supplemental Information 4 — A:Total ion flow chart for mass spectrometry analysis of mixed samples. B: Multi peak pattern of MRM metabolite detection. [file peerj-12-18049-s004.pdf]

The following are the materials, images, and data records for coconut CT testing and metabolite testing. Specifically, as follows:

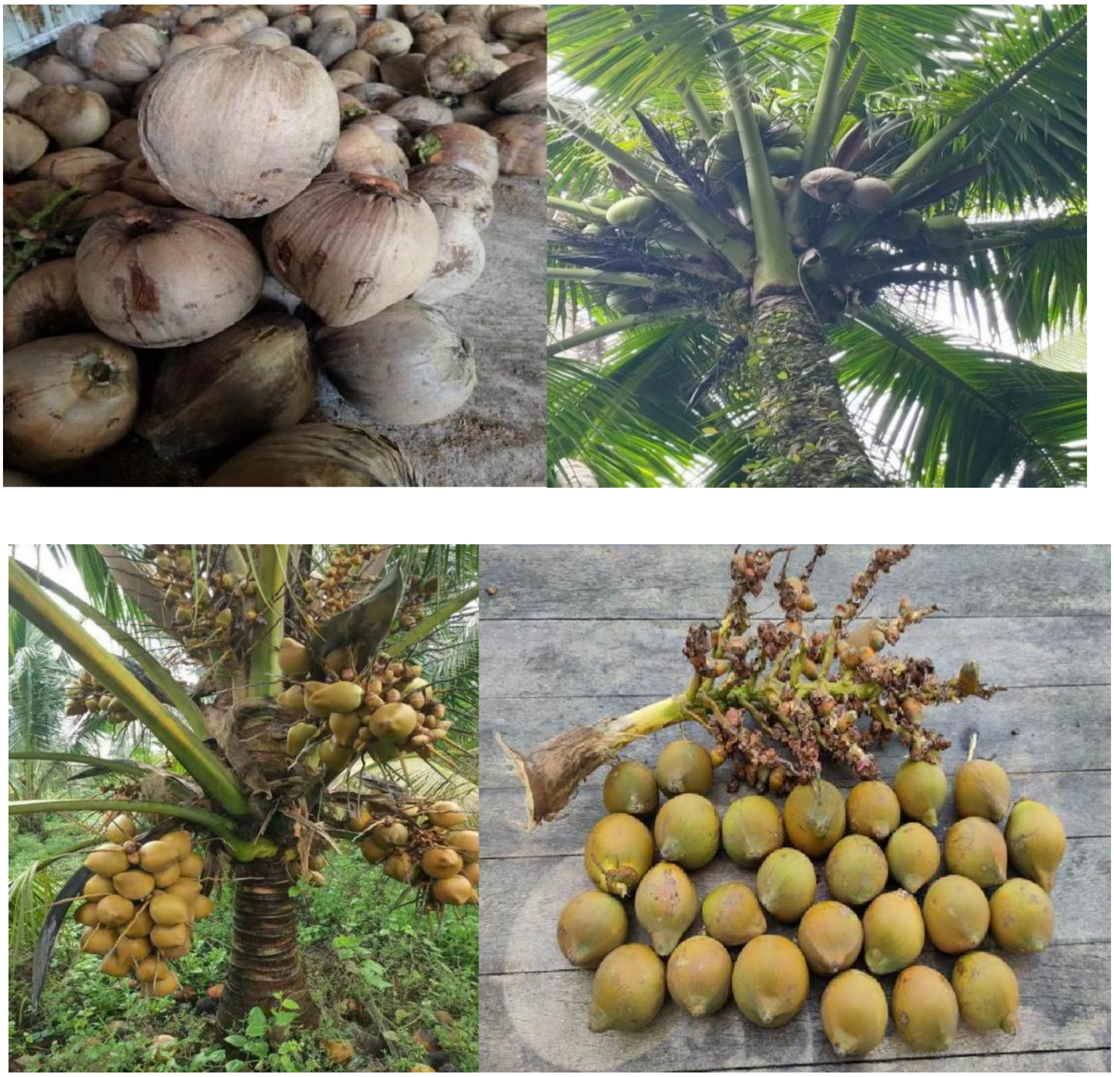

Figure S1: Coconut material tested

Photographer: Chengxu Sun

**Wen Ye 5 Coconut Variety**	
Fruit age	CT images
2-month fruit age	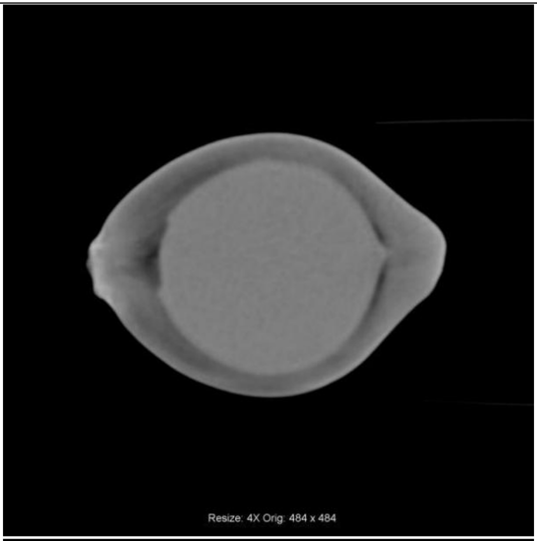
4-month fruit age	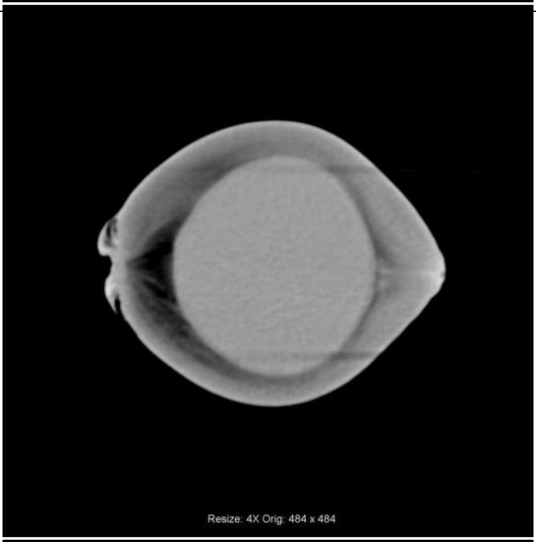
6-month fruit age	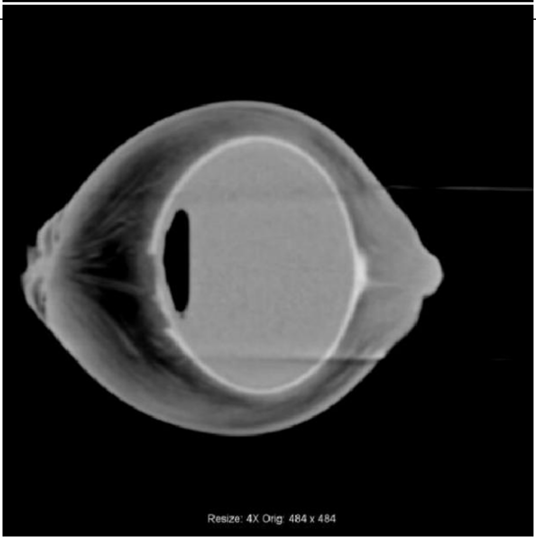

8-month fruit age	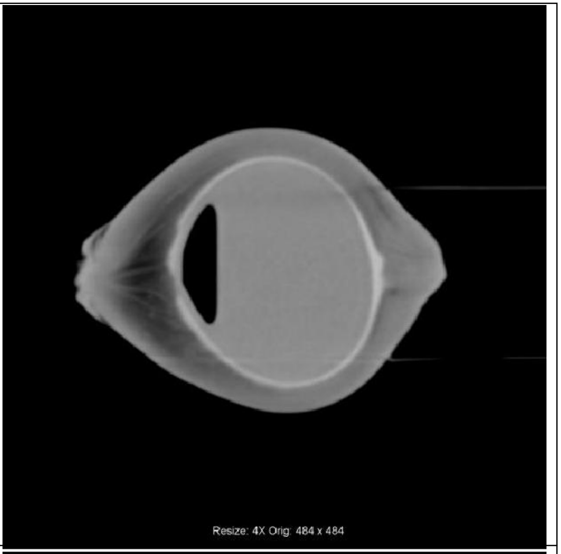
10-month fruit age	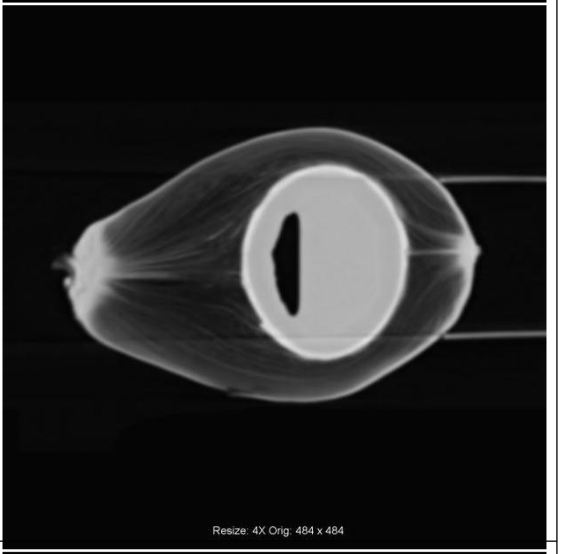
12-month fruit age	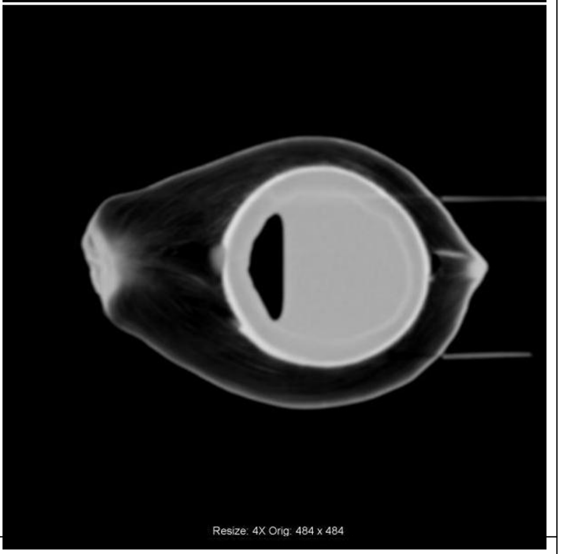

**Hainan Local Coconut**	
2-month fruit age	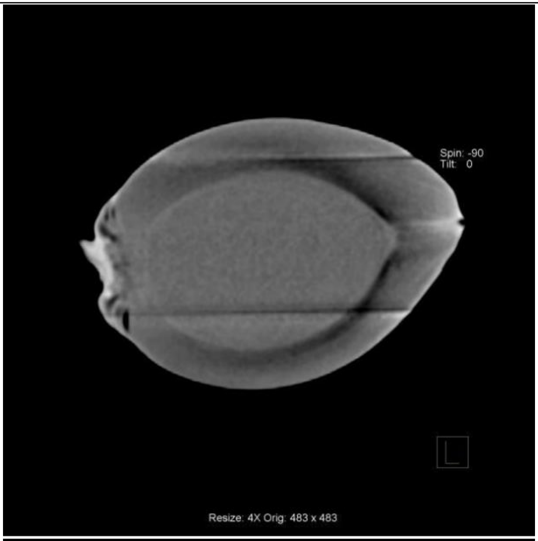
4-month fruit age	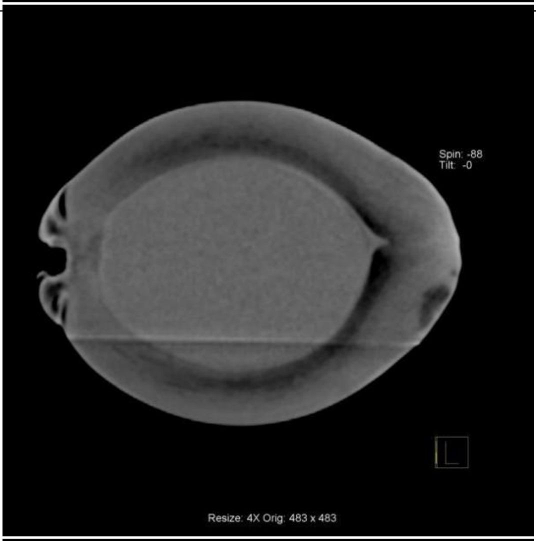
6-month fruit age	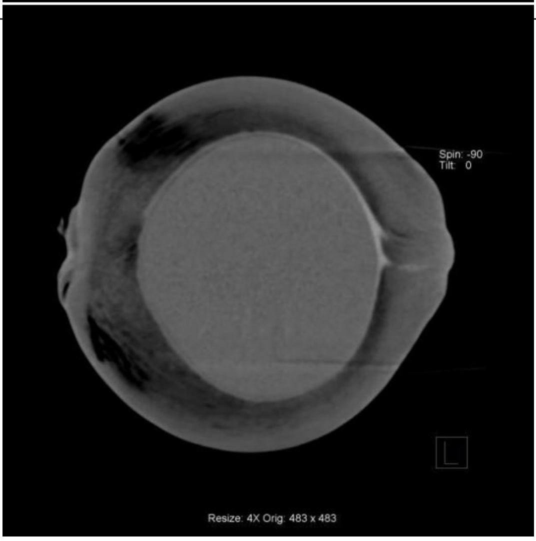

8-month fruit age	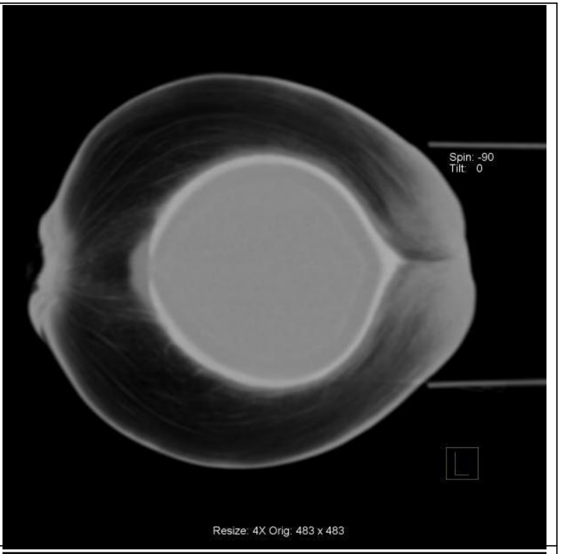
10-month fruit age	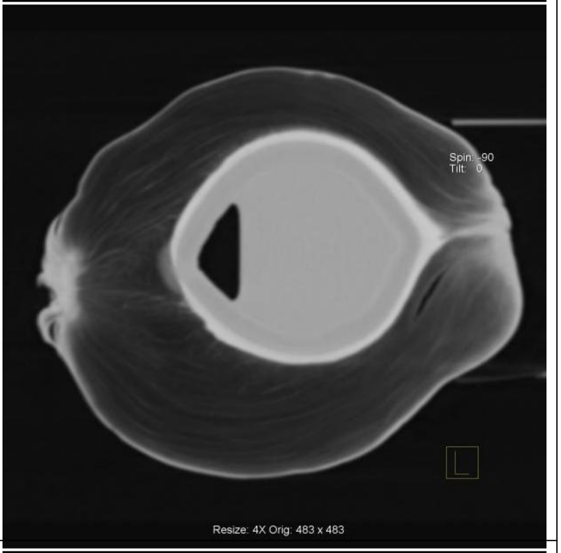
12-month fruit age	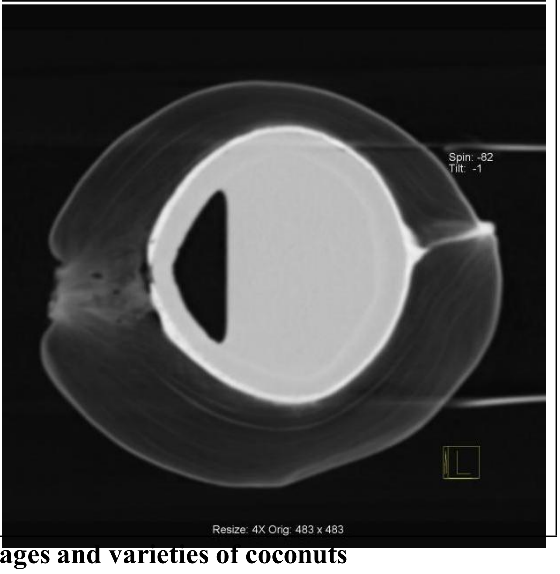

Figure S2 CT images of different developmental stages and varieties of coconuts

Photographer: Chengxu Sun

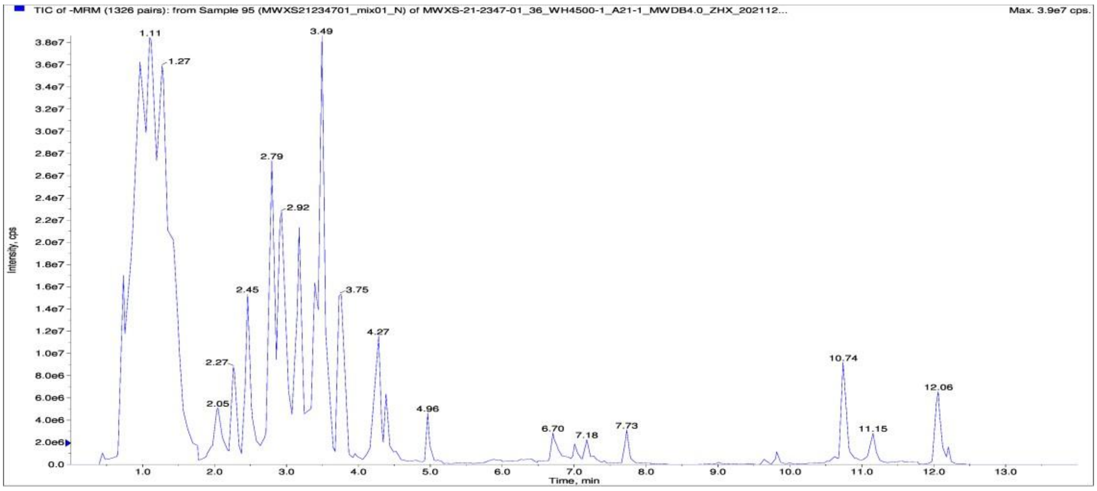

Figure S3 Total ion flow chart for mass spectrometry analysis of mixed samples

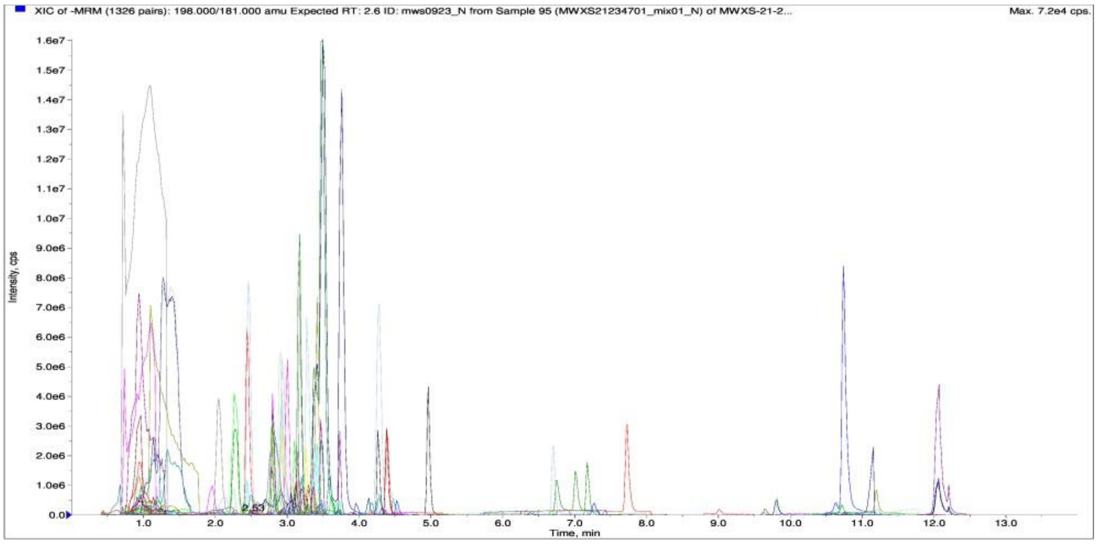

Figure S41 Multi peak pattern of MRM metabolite detection

Table S1 CT test coconut data record table

CT value record table for various tissues of coconut variety "Wenye 5"												
ID	Coconut meat			Coconut shell (inner skin)			Coconut fiber (mesocarp)			Coconut water		
	1	2	3	1	2	3	1	2	3	1	2	3
w5-2-4				29	28	33	-15	-11	-8	26	27	23
w5-2-5				32	33	38	-30	-9	-6	25	24	21
w5-4-7				27	26	32	-10	9	-16	13	16	25
w5-4-8				20	30	37	-37	-15	-21	14	24	16
w5-4-9				26	29	37	-40	-30	-31	20	21	24
w5-6-4	39	40	55	115	107	105	-87	-85	-53	11	19	17
w5-6-5	42	55	56	165	139	165	-59	-63	-77	28	23	22
w5-8-4	43	52	33	103	123	115	-77	-43	-21	28	33	32
w5-8-5	47	57	43	116	136	142	-101	-98	-67	23	17	29
w5-10-1	36	38	41	248	186	202	-828	-814	-775	29	26	26
w5-10-4	30	32	33	220	136	219	-827	-803	-725	19	25	28
w5-12-4	18	45	22	172	155	174	-812	-824	-788	16	14	19
w5-12-5	24	16	53	149	127	123	-785	-714	-809	19	20	9
w5-12-6	26	57	61	125	177	76	-742	-726	-697	15	29	28

CT value record table for various tissues of Local Coconut																				
Identifier	Coconut meat					Coconut shell (inner skin)					Coconut fiber (mesocarp)					Coconut water				
	1	2	3	4	5	1	2	3	4	5	1	2	3	4	5	1	2	3	4	5
CK-2-1						23	24	37	22	25	21	-6	1	15	-14	15	24	20	22	14
CK-2-3						32	28	38	31	24	-11	-10	15	15	-18	24	18	28	22	23
CK-4-1						12	25	14	10	19	-2	1	7	-34	-79	21	22	18	10	28
CK-4-2						32	5	15	17	18	-12	11	22	-14	-53	20	14	29	32	10
CK-6-1						22	99	33	52	-1	-69	-100	5	-34	-88	21	21	29	16	21
CK-6-3						15	77	87	18	-51	-200	-48	-181	-7	-27	30	31	4	9	28
CK-8-1	12	11	2	0	8	225	213	214	221	223	-855	-797	-854	-705	-787	28	24	27	19	28
CK-8-3	4	15	20	13	8	220	238	223	217	195	-780	-721	-801	-699	-605	22	27	25	32	18
CK-10-1	4	-14	1	-5	12	208	242	220	242	247	-767	-851	-871	-820	-823	30	34	30	30	17
CK-10-3	-13	7	-3	-17	4	242	233	212	215	235	-741	-808	-864	-838	-724	30	29	31	12	34
CK-12-1	8	2	14	12	2	201	210	226	277	224	-846	-837	-844	-877	-868	26	29	29	25	27
CK-12-3	15	8	11	13	-3	215	134	190	233	238	-910	-877	-805	-893	-888	13	16	32	18	24

Table S2 Statistical table for the number of differential metablities

Table: Statistical table for the number of differential metabolites

group name	All sig diff	down regulated	up regulated
CK10_vs_W5-10	304	199	105
CK12_vs_W5-12	252	83	169
CK2_vs_W5-2	319	121	198
CK4_vs_W5-4	240	74	166
CK6_vs_W5-6	232	124	108
CK8_vs_W5-8	266	201	65
CK_vs_W5	92	54	38
