# Supplementary material for: Preliminary study on the association between lignan metabolites and CT non-destructive testing of coconut fruit at different developmental stages
Source: PeerJ. 2024 Sep 26;12:e18049. doi: 10.7717/peerj.18049 (PMC11439403; doi:10.7717/peerj.18049)
Supplement: Supplemental Information 5 [file peerj-12-18049-s005.docx]

Rebuttal Letter

Dear Editor,

Thank you for your kind clarification. We appreciate the opportunity to confirm the author list for our manuscript.

To clarify, Mingming Hou is not a new author, and her name was already included in the author list during the initial manuscript submission. In fact, there are no new authors to add to the list. The final and complete author list for our manuscript is as follows:

1.	Chengxu Sun

2.	Xuejing Ma

3.	Jerome Jeyakumar John Martin

4.	Hongxing Cao

5.	Yu Zhang

6.	Yanming Gao

7.	Chunyu Xing

8.	Mingming Hou

We confirm that this is the final and accurate author list for our manuscript. We appreciate your understanding and look forward to the continuation of the peer-review process.

Thank you for your time and consideration.

Best regards,

Jerome Jeyakumar
